# Deletion of *meso*-2,3-butanediol dehydrogenase gene *bud*C for enhanced *D*-2,3-butanediol production in *Bacillus licheniformis*

**DOI:** 10.1186/1754-6834-7-16

**Published:** 2014-01-29

**Authors:** Gaofu Qi, Yanfang Kang, Lu Li, Aifang Xiao, Shumeng Zhang, Zhiyou Wen, Dihong Xu, Shouwen Chen

**Affiliations:** 1 State Key Laboratory of Agricultural Microbiology, College of Life Science and Technology, Huazhong Agricultural University, Wuhan 430070, China; 2 Department of Food Science and Human Nutrition, Iowa State University, Ames, IA 50011, USA; 3 College of Food Science and Technology, Huazhong Agricultural University, Wuhan 430070, China

**Keywords:** Bacillus licheniformis, *D*-2,3-butanediol, *Bud*C gene, *meso*-2,3-butanediol dehydrogenase

## Abstract

**Background:**

*D*-2,3-butanediol has many industrial applications such as chiral reagents, solvents, anti-freeze agents, and low freezing point fuels. Traditional *D*-2,3-butanediol producing microorganisms, such as *Klebsiella pneumonia* and *K. xoytoca*, are pathogenic and not capable of producing *D-*2,3-butanediol at high optical purity. *Bacillus licheniformis* is a potential 2,3-butanediol producer but the wild type strain (WX-02) produces a mix of *D*- and *meso*-type isomers. *BudC* in *B. licheniformis* is annotated as 2,3-butanediol dehydrogenase or acetoin reductase, but no pervious experiment was performed to verify this hypothesis.

**Results:**

We developed a genetically modified strain of *B. licheniformis* (WX-02 Δ*bud*C) as a *D*-2,3-butanediol producer with high optimal purity. A marker-less gene deletion protocol based on a temperature sensitive knock-out plasmid T2-Ori was used to knock out the *bud*C gene in *B. licheniformis* WX-02. The *budC* knock-out strain successfully abolished *meso*-2,3-butanediol production with enhanced *D*-2,3-butanediol production. No *meso*-BDH activity was detectable in cells of this strain. On the other hand, the complementary strain restored the characteristics of wild strain, and produced *meso*-2,3-butanediol and possessed *meso*-BDH activity. All of these data suggested that *bud*C encoded the major *meso*-BDH catalyzing the reversible reaction from acetoin to *meso*-2,3-butanediol in *B. licheniformis*. The *bud*C knock-out strain produced *D-*2,3-butanediol isomer only with a high yield of 30.76 g/L and a productivity of 1.28 g/L-h.

**Conclusions:**

We confirmed the hypothesis that *bud*C gene is responsible to reversibly transfer acetoin to *meso*-2,3-butanediol in *B. licheniformis*. A mutant strain of *B. licheniformis* with depleted *bud*C gene was successfully developed and produced high level of the *D*-2,3-butanediol with high optimal purity.

## Background

*D*-2,3-butanediol as one of the promising bulk chemicals has extensive applications in cosmetics, foods, transport fuels, medicines, and polymers industries [1]. In general, 2,3-butanediol exists in three stereoisomeric forms: *D*-2,3-butanediol, *L*-2,3-butanediol and *meso*-2,3-butanediol [2]. All these isomers are valuable chemicals that provide chiral groups in drugs [3]. *D-*2,3-butanediol is also used as an antifreeze agent because of its low freezing point (-60°C) [1]. The production of 2,3-butanediol with high optical purities is therefore highly desirable [4,5].

Although many microorganisms are capable of synthesizing 2,3-butanediol, the production processes are hindered by various limitations. For example, traditional 2,3-butanediol producing microorganisms, such as *Klebsiella pneumonia* and *K. xoytoca*, are pathogenic [2,6] and produce a mixture of *meso-* and *L*-isomers with low yield and productivity [2,7]. Non-pathogenic species such as *Paenibacillus polymyxa* can produce *D*-2,3-butanediol with a high (up to 98%) enantioselective purity; however, the cell density and the overall *D*-2,3-butanediol productivity is low as the cells need to be grown in micro-aerobic conditions [1,3]. The growth of *P. polymyxa* also needs yeast extract and tryptone, which increases the medium cost and the production recovery cost [8]. *Bacillus licheniformis*, which is a generally-regarded-as-safe (GRAS) organism, is also capable of producing 2,3-butanediol at the industrial level [6,9,10]; however, the wild-type *B. licheniformis* produces a mix of *D*- and *meso-*2,3-butanediol isomers [6].

**Figure 1:**
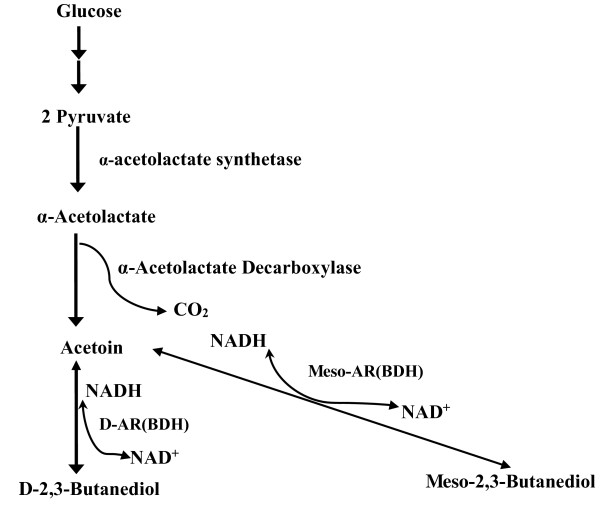
**Metabolic pathways to acetoin and 2,3-butanediol optical isomers in *B. licheniformis*.** AR, acetoin reductase (forward reaction); BDH, 2,3-butanediol dehydrogenase (reverse reaction); NADH, nicotinamide adenine dinucleotide.

The metabolic pathway from pyruvate to 2,3-butanediol has been well studied in *B. subtilis*. As shown in Figure 1, pyruvate is converted to α-acetolactate, and consequently to acetoin. At high dissolved-oxygen and glucose-rich conditions, acetoin can be further converted into 2,3-butanediol by the enzyme called acetoin reductase (AR). The same protein can catalyze the reverse reaction from 2,3-butanediol to acetoin as well, when dissolved oxygen is limited and glucose is depleted. In this case, however, the enzyme is called 2,3-butanediol dehydrogenase (BDH) [1,11]. The meso-AR/BDH is encoded by the *bdh*A gene in *B. subtilis,* therefore, modification of the *bdh*A gene may be an efficient way to increase the optical purity of *D*-2,3-butanediol while avoiding the formation of *meso*-2,3-butanediol by *B. subtilis*[1].

Research has been attempted to produce 2,3-butanediol with high optical purity using genetically engineered microorganisms. For example, Nielsen *et al*. [12] introduced the acetoin and *meso*-2,3-butanediol biosynthesis pathway in *Escherichium coli* by co-expression of 2,3-butanediol dehydrogenase originally derived from yeast, resulting in 1.12 g/L *meso*-2,3-butanediol [12]. Li *et al*. [13] also transferred the gene encoding 2,3-butanediol dehydrogenase from *Enterobacter cloacae* to *E. coli* for expressing *L*-2,3-butanediol from diacetyl with concentrations of 16.1 g/L and 26.8 g/L of *L*-2,3-butanediol produced in batch and fed-batch fermentation, respectively. Although production of high optical purity of 2,3-butanediol isomers has been achieved in engineered *E. coli*, the product yield was usually very low, mainly due to the weak overflow of the metabolic pathway in *E. coli* cells.

Compared to *E. coli, Bacillus* species such as *B. licheniformis, B. subtilis, and B. amyloliquefaciens* have a strong overflow metabolic pathway from glucose. Therefore, modification of the metabolic pathway of *Bacillus* species provides a promising way for producing pure 2,3-butanediol isomer with high product titer. In our previous work, we have isolated a strain of *B. licheniformis* (termed WX-02), which showed a rapid growth and capability of producing γ-poly-glutamic acid (γ-PGA) accompanied with 2,3-butanediol and acetoin [14]. Similar to *B. subtilis* and other *B. licheniformis* strains, however, *B. licheniformis* WX-02 produces a mixture of *D*-2,3-butanediol and *meso*-2,3-butanediol [11,14,15]. Furthermore, the genome of *B. licheniformis* WX-02 was sequenced and the data submitted [GenBank: AHIF01000000], but a gene similar to the *bdh*A gene in *B. subtilis* was not found in *B. licheniformis* WX-02. The gene *bud*C (gene ID: 3100198) in *B. licheniformis* WX-02 genome is annotated as AR; this gene (*bud*C) is the same as that of *B. licheniformis* ATCC 14580 (DSM 13) [16], although it has little similarity (1.67% identity aligned by UniProt (http://www.uniprot.org/?tab=align)) to *bdh*A in *B. subtilis*. The cell extract of *B. licheniformis* also shows AR (BDH) activity, with acetoin, *D*-2,3-butanediol and *meso*-2,3-butanediol also being identified. All these results indicate the existence of the gene encoding AR (BDH) in *B. licheniformis*[17,18]. Recent research by Li *et al*. [10] also shows that the recombinant *E. coli* containing the BDH and glycerol dehydrogenase (GDH) encoding gene from *B. licheniformis* exhibited *meso*-BDH and *D*-BDH activity *in vitro*[10]. The objective of this work was to investigate the specific function of *bud*C in the metabolism of acetoin and 2,3-butanediol in *B. licheniformis* WX-02, followed with developing a strategy of knocking out the *bud*C gene so the production of the sole *D*-2,3-butanediol isomer can be achieved.

## Results

### Establishment of the budC gene knock-out strain and complementary strain

**Figure 2:**
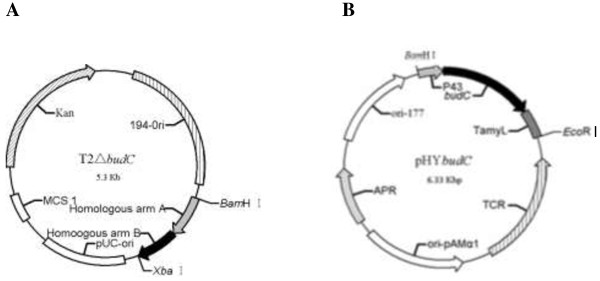
**Construction of recombinant vectors. (A)** Recombinant vector T2Δ*bud*C for *bud*C knock-out. The vector contained a temperature-sensitive replicon from *B. subtilis* (194-Ori), Kanamycin-resistant gene (Kan), and the homologous arm A and B for homologous recombination. **(B)** Recombinant vector of pHY*bud*C for expression of *bud*C in *B. licheniformis*. The vector contained *bud*C expression cassette including P43 promoter, the *bud*C gene and the α-amylase gene (*amy*L) transcription terminator of *B. licheniformis* WX-02.

Figure 2 shows the plasmids for deletion of the *bud*C gene and for construction of the complementary strain. These two plasmids were used for transformation of *B. licheniformis* WX-02 for making the *bud*C deletion mutant and its complementary strain, respectively. The positive strains were then verified by PCR and sequencing.

**Figure 3:**
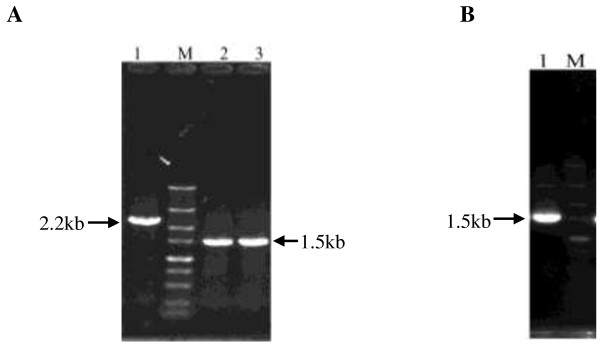
**PCR verification of recombinant strains. (A)** PCR verification of *bud*C knock-out strain. M, DL5000 marker (5,000, 3,000, 2,000, 1,500, 1,000, 750, 500, 250 and 100 bp, up to bottom); lane 1, negative control (PCR product from the genomic DNA of *B. licheniformis* WX-02); lanes 2 and 3, fragment amplified from the genomic DNA of *B. licheniformis* WX-02Δ*budC*. **(B)** PCR verification of complementary strain of *B. licheniformis* WX-02 Δ*bud*C/pHY*bud*C. M, DL5000 marker; lane 1, PCR product of fusion fragment of P43-*bud*C-TamyL from recombinant strain *B. licheniformis* WX-02 Δ*budC*/pHY*budC*.

The PCR results for verification of *bud*C knock-out are shown in Figure 3A. The PCR product amplified from the genomic DNA of *bud*C knock-out strain was about 1,500 bp; while a DNA fragment of about 2200 bp containing the *bud*C gene and its up- and down- stream sequences was amplified from the genomic DNA of wild strain WX-02 by using primers of Δ*budC*-F and Δ*budC*-R as negative control. The PCR product from *bud*C knock-out strain was purified and sequenced, and no other mutation than the *bud*C deletion was found (data not shown), suggesting the successful construction of the *bud*C deficient strain (WX-02 Δ*bud*C). The complementary strain of WX-02 Δ*bud*C (terms as WX-02 Δ*bud*C/pHY*bud*C), was also verified by PCR. As shown in Figure 3B, a DNA fragment of about 1500 bp was amplified from the recombinant plasmid of transformant with a matched size to the fusion fragment of P43-*bud*C-T*amy*L, and then further verified by DNA sequencing (data not shown).

### Effect of budC knock-out on meso-AR/meso and BDH activities

**Figure 4:**
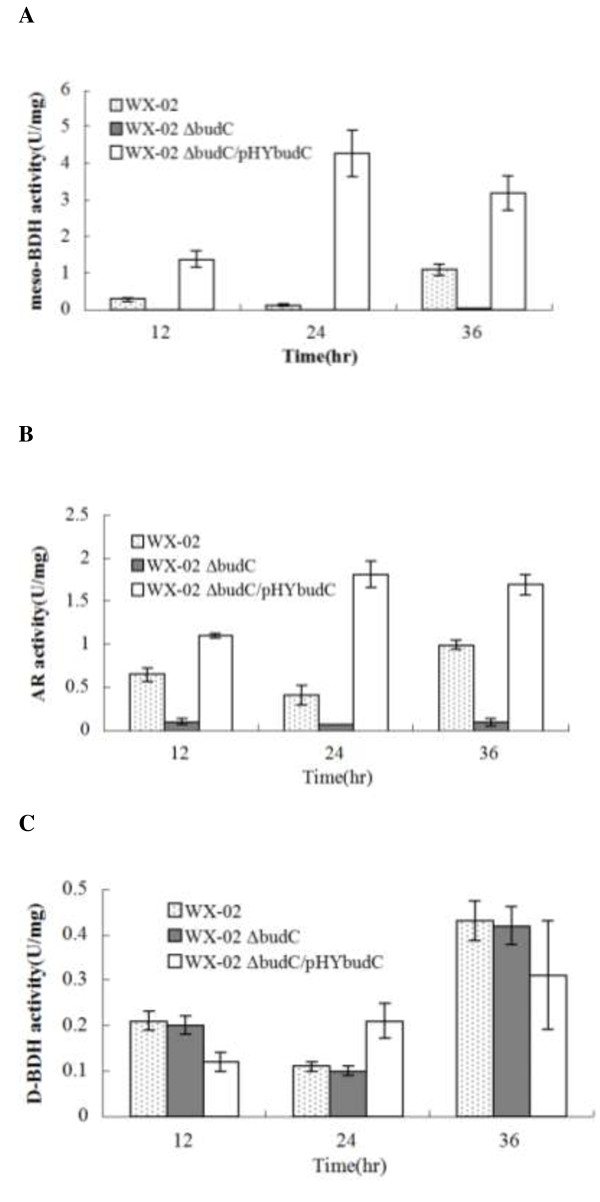
**Activity of 2,3-butanediol dehydrogenas (BDH) and acetoin reductase (AR) in different *B. licheniformis*. (A)***meso*-BDH activity; **(B)** AR activity; **(C)***D*-BDH activity. The data are expressed as mean ± standard error of three replicates.

As described in Figure 1, the enzyme catalyzing the conversion between acetoin and 2,3-butanediol exhibits two activities depending on the culture conditions: AR activity for reduction of acetoin to 2,3-butanediol and BDH activity for dehydrogenation of 2,3-butanediol to acetoin. To investigate the effect of *bud*C knock-out on AR and BDH activities, strains WX-02, WX-02 Δ*bud*C and WX-02 Δ*bud*C/pHY*bud*C were cultured for 12, 24 and 36 h; the specific activities of *meso*-BDH, AR, and D-BDH in the cell extracts were analyzed. As shown in Figure 4A, no *meso*-BDH activity was detected in the cell extracts of WX-02 Δ*budC* throughout the culture; whereas both WX-02 and WX-02 Δ*bud*C/pHY*bud*C exhibited a high *meso*-BDH activity. As for the AR activity, WX-02 Δ*bud*C exhibited a very weak AR activity as compared to the other two strains (WX-02 and WX-02 Δ*bud*C/pHY*bud*C) (Figure 4B). Figure 4C shows that *D*-BDH activity of WX-02 Δ*bud*C was comparable to that of WX-02 and WX-02 Δ*bud*C/pHY*bud*C (Figure 4C). Collectively, the above results indicate that the deletion of the *bud*C gene had a significant effect on *meso*-BDH, but not on *D*-BDH activity, indicating that the *bud*C gene encodes *meso*-BDH but not *D*-BDH. It should also be noted that WX-02 Δ*bud*C/pHY*bud*C restored both *meso*-AR and *meso*-BDH activities compared to the budC gene knock-out strain; these two enzyme activities in WX-02 Δ*bud*C/pHY*bud*C were higher than those in WX-02 (Figure 4A and B). The reason may be due to the multicopy of the *bud*C gene controlled by a strong promoter of P43 in WX-02 Δ*bud*C/pHY*bud*C strain as compared to the wild-type strain [19].

### Effects of budC deletion on 2,3-butanediol configurations

Among the three stereoisomers of 2,3-butanediol, *D*- and *L*-types are racemic and can only be separated in a chiral column; they can be easily separated from the *meso*-type by ordinary non-chiral gas chromatograph (GC) capillary columns [4]. In this study, therefore, *D*-2,3-butanediol and *meso*-2,3-butanediol produced by *B. licheniformis*[2,17] were separated by ordinary non-chiral GC.

**Figure 5:**
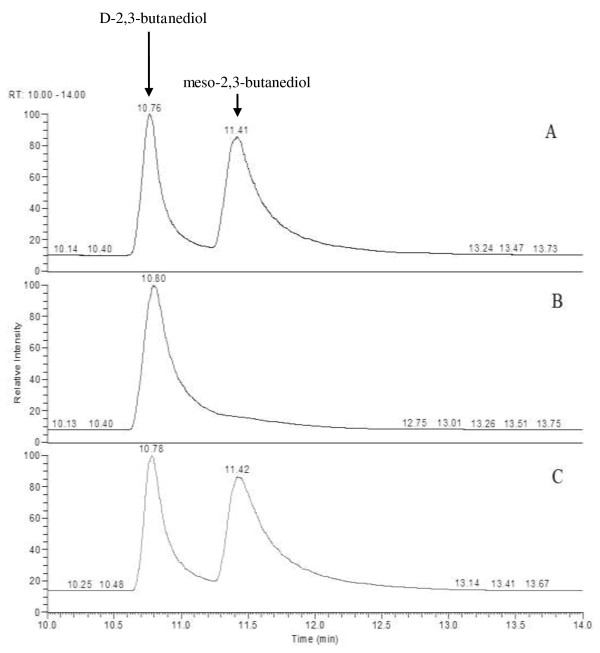
**Chromatograph profile of 2,3-butanediol produced from different *B. licheniformis *strain cultures. (A)** WX-02; **(B)** WX-02 Δ*bud*C; **(C)** WX-02 Δ*bud*C/pHY*bud*C*.*

Strains WX-02, WX-02 Δ*bud*C and WX-02 Δ*bud*C/pHY*bud*C were respectively cultured for 24 h and then 2,3-butanediol in broth was determined. As shown in Figure 5, *D*-2,3-butanediol and *meso*-2,3-butanediol were well-separated. WX-02 *Δbud*C produced *D*-2,3-butanediol but no *meso*-2,3-butanediol, whereas WX-02 and WX-02 *Δbud*C/pHY*bud*C generated both *D*-2,3-butanediol and *meso*-2,3-butanediol (Figure 5). The result clearly shows that the synthesis of *meso*-2,3-butanediol in *bud*C knocked-out strain (WX-02 Δ*bud*C) was successfully deleted, whereas the complementation of the *budC* knock-out strain (WX-02 Δ*bud*C/pHY*bud*C) restored the capability of *meso*-2,3-butanediol synthesis. The result confirmed that the *bud*C gene was responsible for *meso*-2,3-butanediol production in *B. licheniformis*. Unexpectedly, the transformed strain (WX-02 Δ*bud*C/pHY*bud*C) had a higher *meso*-BDH activity, but this strain did not produce more *meso*-2,3-butanediol than the wild-type (WX-02). We believe that the synthesis of *meso*-2,3-butanediol in this transformant may be controlled by other rate-limiting factors. For example, the conversion from acetoin to *meso*-2,3-butanediol also needs nicotinamide adenine dinucleotide (NADH) as the electro-donor [6,18]; this NADH in the transformant may be the controlling factor, although the activity of *meso*-BDH is higher in the strain.

### Production of 2,3-butanediol and acetoin by budC knock-out strain and wild-type strain

**Figure 6:**
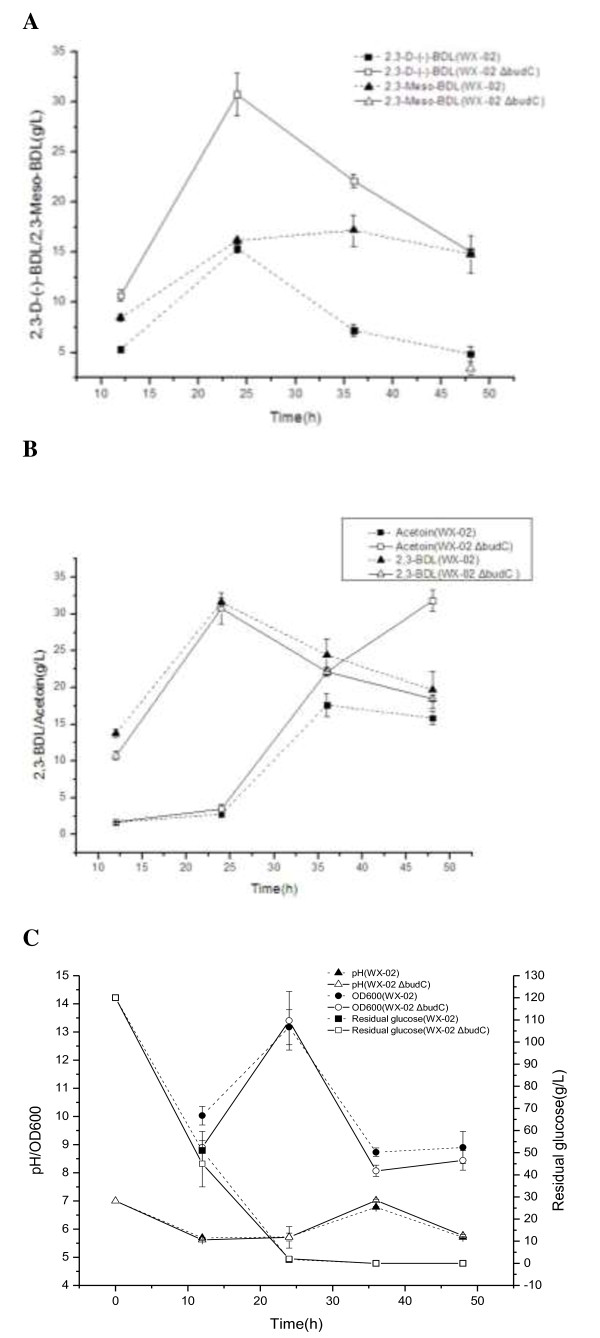
**Comparison of metabolites production, cell growth, and glucose consumption profile by wild-type (WX-02) and *bud*C knock-out (WX-02 Δ*bud*C) *B. licheniformis *strains. (A)***D*- and *meso*-2,3-butanediol production. **(B)** Total 2,3-butanediol and acetoin production. **(C)** Cell density, medium pH, and residual glucose concentration. Data are expressed as mean ± standard errors of three replicates. BDL, butanediol.

The production profile of 2,3-butanediol and acetoin by the wild strain and *budC* knock-out strain are presented in Figure 6. As shown in Figure 6A, wild-strain WX-02 produced both *D*- and *meso*-types of 2,3-butanediol throughout the culture. The concentration of these two isomers increased in the first 24 h; beyond this culture time, *D*-2,3-butanediol concentration decreased whereas *meso*-2,3-butanediol leveled off in the remainder of the culture period. For the culture of WX-02 Δ*bud*C strain, however, only the *D*-type of 2,3-butanediol was produced throughout all the culture. The *D*-2,3-butanediol concentration increased for the first 24 h, and decreased afterwards. Only a trace amount of *meso*-2,3-butanediol was detected at the end of culture (48 h) of WX-02 Δ*bud*C. Throughout all the culture period, the concentration of *D*-2,3-butanediol in the WX-02 Δ*bud*C culture was approximately the summation of the *D*- and *meso*-2,3-butanediol isomers in the WX-02 culture, indicating the *meso*-2,3-butanediol originally formed in the wild-type cells switched to additional *D*-2,3-butanediol in the WX-02 Δ*bud*C mutant. The above production profiles for *D*- and *meso*-type of 2,3-butanediol were also reported in the culture of *B. subtilis* and *Serratia marcescens*[9,20].

As for the production of acetoin and total 2,3-butanediol, Figure 6B shows that for both the wild-type and *bud*C gene knock-out strain, total 2,3-butanediol production increased rapidly in the first 24 h and gradually decreased afterwards; concurrently, the acetoin production of the two strains was low in the first 24 h, but increased rapidly from 24 to 36 h. The loss of the *bud*C gene in the WX-02 Δ*bud*C strain resulted in more acetoin accumulation than wild strain after 36 h.

Figure 6C shows pH change, glucose utilization, and biomass density of the two strain cultures. The pH values were low in the first 24 h, indicating the synthesis of organic acids by the strains. This low pH level favored the synthesis of 2,3-butanediol (Figure 6B). In the later stage of culture, the slight increase in pH favored the conversion of *D*-2,3-butanediol to acetoin, which is evidenced by the increased concentration of acetoin in the medium (Figure 6B). The similar trend between pH and acetoin/2,3-butanediol conversion was also found in *B. subtilis*[21]. Figure 6B also shows that glucose for the two cultures had a similar trend; the glucose was rapidly consumed within the first 24 h, which corresponds to a rapid cell growth in the two cultures. After 24 h the glucose in the medium was almost depleted; as a result the cells of both the wild-type strain and *budC*-gene knock-out mutant ceased growth due to the glucose (Figure 6C). However, the wild-type cells showed a higher cell density than the mutant; the reason was probably due to the consumption of acetoin by the wild-type strain for supporting the cell growth. Indeed, it has been reported that acetoin can be a good carbon source for the culture of *B. licheniformis* once the major carbon source, glucose, is depleted [20,22]. This may also explain why the acetoin concentration in the mutant culture was much higher than that in the wild-type strain (Figure 6B).

## Discussion

As a valuable compound, *D*-2,3-butanediol has been widely used as a major composition in solvents, anti-freeze agents, synthetic rubber, and plastics [2]. It can also be used as a potential fuel with a low freezing point and its heating value is comparable to that of ethanol and methanol [6]. Various efforts have been attempted for producing optical purity of *D*-2,3-butanediol by genetically modified microorganisms; however, the yield of *D*-2,3-butanediol has still been very low. For example, recombinant *E. coli* expressing the enzyme BDH was found to produce 6.1 g/L of *D*-2,3-butanediol [21], and *B. licheniformis* with a deleted lactate dehydrogenase gene (*ldh*) to produce 13.77 g/L of *D*-2,3-butanediol [6]. Heterologous expression of acetoin reductase of *Clostridium beijerinckii* in *C. acetobutylicum* has resulted in a range of 1.8 to 1.98 g/L *D*-2,3-butanediol [7].

The strain *B. licheniformis*, WX-02, used in this study was previously isolated for the production of γ-PGA with 2,3-butanediol and acetoin being the co-products. This strain can grow in a simple medium containing glucose, glutamic acid, and mineral salts [14]. As the strain WX-02 produces mixed stereoisomers of 2,3-butanediol, modification of its metabolic pathway for sole production of pure isomer of *D*-2,3-butanediol is desirable. To date, there have been several challenges to making a recombinant strain of *B. licheniformis*, including low transformation efficiency and a lack of information about the *meso-*BDH encoding gene*.* For example, Wang *et al*. [6] successfully deleted the *ldh* gene from genomic DNA in *B. licheniformis* by transforming protoplasts of the cells with a recombinant knock-out plasmid; however, the designed protoplast system was very complicated and the transformation efficiency was low [6]. In this paper, we successfully transformed *B. licheniformis* WX-02 with a recombinant knock-out plasmid with high efficiency. It demonstrates that T2-ori-based knock-out plasmid and the electro-transformation approach can be used for the metabolic modification of the *B. licheniformis* WX-02 strain for producing pure *D*-2,3-butanediol with high titer.

Previous reports showed that the *bdh*A gene encoding BDH is responsible for catalyzing acetoin to 2,3-butanediol in *B. subtilis*, and the insertion inactivation of *bdh*A completely blocks 2,3-butanediol synthesis [11]. For *B. licheniformis*, however, no *bdh*A gene was found in the genome*.* In the attempt for identifying the gene responsible for catalyzing acetoin to 2,3-butanediol in *B. licheniformis,* Wang *et al*. [6] reported the depletion of the *ldh* gene for the production of high optical purity of *D*-2,3-butanediol in *B. licheniformis* with 13.77 g/L *D*-2,3-butanediol being produced in optimized conditions [6]. However, our previous study in knocking out the *ldh* gene in *B. licheniformis* WX-02 to block lactate accumulation resulted in reduced acetoin and 2,3-butanediol production (unpublished data). The recent report showed that the *bud*C gene might be the gene encoding *meso*-BDH in *B. licheniformis* according to the detectable enzyme activity of the recombinant protein of *bdh* (same as *bud*C) gene by *E. coli*[10]. Therefore, we hypothesized that the *bud*C gene is responsible for catalyzing acetoin to *meso*-2,3-butanediol in *B. licheniformis.*

Our previous research shows that the *bud*C gene in *B. licheniformis* might be annotated as BDH for this species [16]*.* In this work, we confirmed the *bud*C in *B. licheniformis* as the gene encoding *meso*-BDH for the reversible reaction from acetoin to *meso*-2,3-butanediol [18], based on the fact that the deletion of *bud*C gene in *B. licheniformis* WX-02 completely blocked *meso*-2,3-butanediol production with significant enhanced production of *D*-2,3-butanediol (Figure 6A). However, the BudC protein sequence [NCBI: YP_006713433.1], aligned by blastP in the NCBI non-redundant protein database, showed that only one protein from *B. sonorensis* annotated as 2,3-BDH, AR or diacetyl reductase was similar (*E*-value <8e-112) to BudC protein. Moreover, the BudC protein sequence has a very low identity with the BDH found in other *Bacillus* species. For example, it has only 11.67%, 9.98% and 11.58% similarity to the BDHs from *B. subtilis* 168 (NP_388505.1), *B. cereus* YUF-4 (BAB60856.1) and *B. amyloliquefaciens* DSM 7 (YP_003919213.1), respectively. All these results indicate that unique BDH-encoding gene in *B. licheniformis* is different from other *Bacillus* genus.

Although the deletion of *bud*C gene caused a slight decrease (about 5 to 10%) in cell growth (Figure 6C), it significantly enhanced the *D*-2,3-butanediol production (Figure 6C) (30.76 g/L) compared to both the wild strain in this work and the genetically modified strain with the deletion of the *ldh* gene (13.77 g/L) [6]. Finally, it should be noted that even when *bud*C was deleted from the *B. licheniformis* WX-02 genome, there was still a small amount of *meso*-2,3-butanediol found at the end of fermentation period (Figure 6A). This may be due to the existence of other genes encoding minor *meso*-BDHs, or acetylacetoin reductase catalyzing acetoin to *meso*-2,3-butanediol [18]. Indeed, low concentration of glucose and high concentration of acetoin, as found in this work (Figure 6A and C), can induce acetylacetoin synthase to transform acetoin to *meso*-2,3-butanediol through the 2,3-butanediol cycle [18].

## Conclusions

In summary, this report revealed the specific function of *bud*C for the transformation between acetoin and 2,3-butanediol in *B. licheniformis*. The *D*-2,3-butanediol production level obtained in this work was the highest among the reported *Bacillus* genus. The study provides a deep understanding of acetoin and 2,3-butanediol metabolism in *B. licheniformis*, and a possible way for enhancing the production of pure *D*-2,3-butanediol isomer through genetic modification.

## Materials and methods

### Cell strain, plasmids, primers and growth media

Experiments were performed with the strains and plasmids listed in Table 1. The oligonucleotide primers listed in Table 2 were designed on the basis of *B. licheniformis* WX-02 genome sequence [GenBank: AHIF00000000] [16]. Luria-Bertani (LB) medium was prepared for culture of *E. coli* DH5α and also *B. licheniformis*[23]. Medium used for culturing *B. licheniformis* was a slight modification of that described in a previous report [24], consisting of (per liter) 120 g glucose, 33 g corn steep liquor, 9.00 g (NH_4_)_2_SO_4_, 1.00 g K_2_HPO_4_, 1.50 g MgSO_4_, 0.50 g NaCl, 0.12 g ZnCl_2_, 1 mg FeCl_3_, and 1 mg MnSO_4._ The medium was adjusted to 7.0 before autoclaving at 121°C for 15 minutes.

**Table 1:** Bacterial strains and plasmids used in this study

**Strains and plasmids**	**Characteristics**^**a**^	**Source or reference**
*E. coli* strains		
DH5α	F^–^ Φ80d/*lac*ZΔM15, Δ(*lacZYA-argF*) U169, *recA*1, *endA*1, *hsdR*17 (*r*_K_^–^, *m*_K_^+^), *phoA*, *supE*44, λ^–^, *thi*-1, *gyrA*96, *relA*1	Laboratory stock
*B. licheniformis* strains		
WX-02	CCTCC M208065, wild type	Laboratory stock [14]
WX-02 Δ*bud*C	*bud*C knock-out mutant of WX-02	This study
WX-02 Δ*bud*C/pHY*bud*C	plasmid-based *bud*C complementation strain of WX-02 Δ*bud*C by introduction of pHY*bud*C, Tc^r^	This study
Plasmids		
T2(2)-ori	*E. coli*-*B. licheniformis* shuttle vector, ori_pUC_/ori_ts_, temperature-sensitive, Kan^r^	Laboratory stock
T2Δ*bud*C	T2(2)-ori derivative containing homologous arms for *bud*C knock-out	This study
pHY300PLK	E. coli-*B. licheniformis* shuttle vector, Ap^r^(*E. coli*), Tc^r^(*E. coli* and *B. licheniformis*)	TaKaRa
pHY*bud*C	pHY300PLK derivative containing *bud*C, P43 promoter and TamyL (amyL terminator), Ap^r^(*E. coli*), Tc^r^(*E. coli* and *B. licheniformis*)	This study

^a^Tc^r^, tetracycline resistance; Ap^r^, ampicillin resistance; ori_ts_, temperature-sensitive *Bacillus* origin of replication; Kan^r^, Kanamycin resistance; CCTCC, China Center for Type Culture Collection.

**Table 2:** Primers used in this study

**Primer name**	^**a**^**Sequence 5′ → 3′**
Δ*budC*-A-F	CGC**GGATCC**AAAGCGCATGTTTTAAAAC
Δ*budC*-A-R	CCGCC*CTCCATATAGAATATAATTTTAAAAATAAACATCTTCTTTCTATAAGTAA*
Δ*budC*-B-F	ACCAA*TTACTTATAGAAAGAAGATGTTTATTTTTAAAATTATATTCTATATGGAG*
Δ*budC*-B-R	GC**TCTAGA**CCTCGCACTAGTGTATTTTGAAAC
Δ*budC*-F	CGAACTCCATGAACTGACAGTC
Δ*budC*-R	TTGCTATTTCCTGTTATGACC
P43-*budC*-TamyL-1	CGC**GGATCC**TGTCGACGTGCATGCAGG
P43-*budC*-TamyL-2	*CAATTTTTCCAGATACTTTACTCATGTGTACATTCCTCTCTTACCTATA*
P43-*budC*-TamyL-3	*TATAGGTAAGAGAGGAATGTACACATGAGTAAAGTATCTGGAAAAATTG*
P43-*budC*-TamyL-4	*CGTCCTCTCTGCTCTTCTATCTTTTAATTAAATACCATTCCGCCATC*
P43-*budC*-TamyL-5	*GATGGCGGAATGGTATTTAATTAAAAGATAGAAGAGCAGAGAGGACG*
P43-*budC*-TamyL-6	CCG**GAATTC**GATCACCCGCGATACCGTC
Δ*budC* A signal crossover-F	CTTCACATGGACGATCCTAAT
Δ*budC* A single crossover-R	TGTTCCTCCGTAAACCGCTAAG
Δ*budC* B single crossover-F	CAACCACCCCTATTGAAAGCAT
Δ*budC* B single crossover-R	GATACCTGTCCGCCTTTCTCC

^a^Restriction sites highlighted in bold. Italics stands for the overlap region for splicing by overlapping extension PCR (SOE-PCR).

### Chemicals and materials for cloning

Acetoin (98%) and 2,3-butanediol (98%) were purchased from Shanghai Jingchun Reagent (China). *D*-2,3-butanediol (>96%) was purchased from Tokyo Chemical Industry (Tokyo, Japan), *meso*-2,3-butanediol (99%) was purchased from Sigma-Aldrich (Sigma, St. Louis, MO, USA). All other chemicals were of analytical grade supplied by Sinopharm Chemical Reagent (Shanghai, China). T4 DNA ligase and DNA marker were purchased from Takara Bio (Dalian, China). TransStart FastPfu DNA Polymerase was purchased from TransGen Biotech (Beijing, China). Plasmid Miniprep Kit was obtained from Zoman Biotech (Beijing, China). Nucleotide sequences were determined by Beijing Genomics institution (Beijing, China).

### Construction of plasmids

*B. licheniformis* WX-02 or *B. subtilis* 168 was cultured in LB medium overnight, and then collected for extraction of genomic DNA based on the method described previously [25]. The extracted genomic DNA was stored at -20°C prior to use. The gene *bud*C was deleted by the double-crossover homologous recombination method with the primers listed in Table 2. First, two homologous arms (homologous to the 5′ and 3′ coding regions of the *bud*C gene) of approximately 500 bp were amplified by PCR from the genomic DNA of *B. licheniformis* WX-02 by primers of Δ*bud*C-A-F and Δ*bud*C-A-R, Δ*bud*C-B-F and Δ*bud*C-B-R, respectively. These two homologous arms were ligated by splicing with overlapping extension PCR (SOE-PCR) with primers of Δ*bud*C-A-F and Δ*bud*C-B-R [6]. The DNA fragment was subcloned in vector T2(2)-ori joined by *Bam*H I and *Xba* I restriction sites. T2(2)-ori was a previously constructed shuttle plasmid for construction of knock-out vector for *B. licheniformis*, with a temperature-sensitive replicon from *B. subtilis* to promote single crossover in bacterial cells [26]. The resulting plasmid was further verified by sequencing. A recombinant vector for *bud*C knock-out was designated as T2Δ*bud*C (Figure 2A).

The fusion of the P43 promoter of *B. subtilis* 168, *bud*C gene of WX-02, and terminator of *amy*L gene of *B. licheniformis* WX-02 were achieved by SOE-PCR with primers of P43-*budC*-TamyL-1 to 6 (Table 2) and templates of genomic DNA from *B. licheniformis* WX-02 or *B. subtilis* 168. Then the DNA fragment amplified by SOE-PCR was cloned into the plasmid of pHY300PLK joined by the *Bam*H I and *Eco*R I restriction sites. The resulting plasmid was verified by sequencing. A recombinant vector for expression of *bud*C in *B. licheniformis* WX-02 was designated as pHY*bud*C (Figure 2B).

### Construction of the budC knock-out strain of WX-02

Competent cells of *E. coli* DH5α and *B. licheniformis* WX-02 were prepared for transformation of constructed plasmids as described previously [23,27]. *E. coli* DH5α was transferred with T2Δ*bud*C plasmid and cultured in LB medium with kanamycin (20 μg/mL). The plasmid T2Δ*bud*C isolated from the recombinant *E. coli* DH5α was used for transforming into *B. licheniformis* WX-02.

*B. licheniformis* WX-02 was electrotransformed with the recombinant T2Δ*budC* plasmid according to the method described previously [27]; the transformants were selected by kanamycin resistance (20 μg/mL) followed with verification by PCR using the primers Δ*bud*C-A-F and Δ*bud*C-B-R (Table 2). The selected positive transformant was cultured in LB medium containing kanamycin (20 μg/mL) at 45°C for 8 h, and the temperature-sensitive replicon of the T2Δ*budC* plasmid did not work at this temperature. Therefore, the high growth temperature promoted the first crossover in the cells. The mutants with kanamycin resistance were selected, and further verified by PCR with primers of Δ*bud*C A single crossover-F and Δ*bud*C A single crossover-R for crossover upstream, or Δ*bud*C B single crossover-F and Δ*bud*C B single crossover-R for crossover downstream. Then the selected colonies with single crossover were picked up and cultured in LB medium at 37°C for 8 hours, this process was repeated six times. After serial transfer without antibiotics, cells were plated on LB agar plates, and then replicated in kanamycin plates for selection of kanamycin-sensitive colonies. The *bud*C knock-out strains that had looped out the kanamycin-resistant gene by the second crossover were selected. The mutant WX-02 Δ*bud*C was confirmed by PCR with primers of Δ*budC*-F and Δ*budC*-R (Table 2) and nucleotide sequencing.

### Construction of the complementary strain of WX-02 ΔbudC

The complementation of *B. licheniformis* WX-02 Δ*bud*C was conducted with a *bud*C expression plasmid. The *B. licheniformis* WX-02 Δ*bud*C was electrotransformed with pHY*bud*C DNA according to the method described previously [27], and the transformants were first selected by LB agar plates with 20 μg/mL tetracycline [27], followed with verification by PCR with primers of P43-*bud*C-TamyL-1 and P43-*bud*C-TamyL-6 (Table 2). The recombinant strain was designated as WX-02 Δ*bud*C/pHY*bud*C.

### Detection of BDH and AR activities in cells

The wild strain WX-02, mutant strain WX-02 Δ*bud*C, and complementary strain WX-02 Δ*bud*C/pHY*bud*C were cultured for 12, 24 and 36 h. The cell extracts from these three cultures were prepared for determining the 2,3-BDH and AR activity based on the previous methods [11]. The reaction system contains 4 mmol/L NAD^+^ and 100 mmol/L 2,3-butanediol for the BDH assay or 0.2 mmol/L NADH and 50 mmol/L acetoin for the AR assay [11]. The cell extracts and reaction system were preheated at 37°C, the 200-μL reaction system was then added to a 96-well UV-star microplate (Greiner Bio-One, Germany) followed with addition of 5 μL cell extracts. The microplate was immediately put into a microplate reader (BioTek, USA) and reacted at 37°C for 5 minutes. Absorbance at 340 nm was measured initially and the end of the reaction. Under these conditions, one unit of BDH or AR activity was defined as 1 μmol of NADH produced or consumed by 1 mg of protein per minute. The protein concentration of cell extracts was determined by the Coomassie brilliant blue method [28].

### Analysis

Cell density was determined by the optical absorbance at 600 nm (OD_600_). The concentration of residual glucose was measured by a biosensor equipped with a glucoseoxidase electrode (SBA-40C, China). Single colonies of the wild strain of WX-02, mutant strain of WX-02 Δ*bud*C, and complementary strain of WX-02 Δ*bud*C/pHY*bud*C on the LB plate were transferred into 250-mL flasks containing 50 mL LB medium and incubated at 37°C for 11 h in an orbital shaker at 180 rpm until the OD_600_ of the culture reached approximately 4.2. The cells were then sub-cultured for 48 h in the same conditions. The samples were collected periodically to determine the time course of cell density, residual glucose, and product (acetoin and 2,3-butanediol) concentrations using previously described methods [29].

Acetoin, *D*-2,3-butanediol, and *meso*-2,3-butanediol were extracted by ethyl acetate and then quantified using Trace GC Ultra Gas Chromatograph (Thermo, USA) equipped with a flame ionization detector and TR-WAX capillary column (30 m × 0.32 mm ID, 0.25 μm film). Nitrogen was used as the carrier gas with a flow rate of 1.0 mL/minute; the injected volume was 1 μL with a splitless injection mode. The injector temperature and the detector temperature were 215°C and 245°C, respectively. The column was maintained at 50°C for 1.5 minutes, increased at a rate of 10°C/minute to 110°C for 0.5 minutes, 5°C/minute to 150°C for 0.5 minutes, and 20°C/minute to 220°C for 1 minute. The concentration of acetoin and 2,3-butanediol was quantified using the internal standard (butanol).

## Abbreviations

Amy: amylase; AR: acetoin reductase; BDH: 2,3-butanediol dehydrogenase; BDL: butanediol; bp: base pairs; GC: gas chromatograph; Ldh: lactate dehydrogenase; NADH: nicotinamide adenine dinucleotide; OD: optical density; SOE-PCR: splicing with overlapping extension PCR; γ-PGA: γ-poly-glutamic acid.

## Competing interests

The authors declare that they have no competing interests.

## Authors’ contributions

GQ conceived of the study, performed the data analysis, and coordinated the manuscript draft and revision. YK and LL executed the experimental work and data analysis. AX and SZ executed the experimental work. ZW helped to revise and proofread the manuscript. DX helped with data analysis. SC conceived the study, and coordinated the manuscript draft and revision*.* All authors read and approved the final manuscript.
